# Restructuring of a Peat in Interaction with Multivalent Cations: Effect of Cation Type and Aging Time

**DOI:** 10.1371/journal.pone.0065359

**Published:** 2013-06-04

**Authors:** Yamuna Kunhi Mouvenchery, Alexander Jaeger, Adelia J. A. Aquino, Daniel Tunega, Dörte Diehl, Marko Bertmer, Gabriele Ellen Schaumann

**Affiliations:** 1 University of Koblenz-Landau, Institute for Environmental Sciences, Department of Environmental and Soil Chemistry, Landau, Germany; 2 University of Leipzig, Institute for Experimental Physics II, Faculty of Physics and Earth Sciences, Leipzig, Germany; 3 University of Natural Resources and Life Sciences, Vienna Institute of Soil Research,Vienna, Austria; Utrecht University, The Netherlands

## Abstract

It is assumed to be common knowledge that multivalent cations cross-link soil organic matter (SOM) molecules via cation bridges (CaB). The concept has not been explicitly demonstrated in solid SOM by targeted experiments, yet. Therefore, the requirements for and characteristics of CaB remain unidentified. In this study, a combined experimental and molecular modeling approach was adopted to investigate the interaction of cations on a peat OM from physicochemical perspective. Before treatment with salt solutions of Al^3+^, Ca^2+^ or Na^+^, respectively, the original exchangeable cations were removed using cation exchange resin. Cation treatment was conducted at two different values of pH prior to adjusting pH to 4.1. Cation sorption is slower (>>2 h) than deprotonation of functional groups (<2 h) and was described by a Langmuir model. The maximum uptake increased with pH of cation addition and decreased with increasing cation valency. Sorption coefficients were similar for all cations and at both pH. This contradicts the general expectations for electrostatic interactions, suggesting that not only the interaction chemistry but also spatial distribution of functional groups in OM determines binding of cations in this peat. The reaction of contact angle, matrix rigidity due to water molecule bridges (WaMB) and molecular mobility of water (NMR analysis) suggested that cross-linking via CaB has low relevance in this peat. This unexpected finding is probably due to the low cation exchange capacity, resulting in low abundance of charged functionalities. Molecular modeling demonstrates that large average distances between functionalities (∼3 nm in this peat) cannot be bridged by CaB-WaMB associations. However, aging strongly increased matrix rigidity, suggesting successive increase of WaMB size to connect functionalities and thus increasing degree of cross-linking by CaB-WaMB associations. Results thus demonstrated that the physicochemical structure of OM is decisive for CaB and aging-induced structural reorganisation can enhance cross-link formation.

## Introduction

Interactions between cations and natural organic matter (NOM) and their significance in the environmental role of cations in soils have been intensively discussed during the last two decades [1]–[7]. However, a recent review demonstrated that some important questions relevant in real soil and peat organic matter remain unresolved [8]. This is mainly because certain interactions like inner sphere complexation or outer sphere complexation may be hampered in soil or peat organic matter if the distance between charged functional groups is too large to be bridged solely by cations [8].

This may lead to a significant amount of outer sphere complexes although inner sphere complexes are thermodynamically more favorable [1], leading to the hypothesis that associations of cations and water molecules can bridge distant functional groups. Consequently, a cross-linked network dependent on moisture conditions [9], [10] similar to that cross-linked by water molecule bridges (WaMB) alone [11]–[13] may form. Thus, three types of cation bridges (CaB) can be distinguished: (1) direct CaB via inner sphere complexes, (2) direct CaB via outer sphere complexes and (3) indirect CaB via WaMB [1], [4], [8], [11], [14]. CaB are expected to increase local rigidity of supramolecular environment [3], [15], [16] and may reduce wettability of organic matter (OM) due to fixation of certain supramolecular orientation [17], [18]. These ideas, however, still require experimental evidence, which implies methodical challenges to be met for the highly complex and heterogeneous OM.

Methods to assess rigidity of supramolecular structures in soil organic matter (SOM) involve thermoanalytical and nuclear magnetic resonance (NMR) spectroscopic methods. Differential scanning calorimetry (DSC) can help to probe matrix rigidity via the temperature of the non-reversing step transition around 40–80°C [12], [19], [20], which has been attributed to the disruption of WaMB. The higher the WaMB transition temperature, the higher is the matrix rigidity in the surroundings of WaMB [4], [12]. Proton NMR wideline spectroscopy in air-dried samples, upon a heating event, gives insight into changes of mobile water and this indicates the amount of WaMB [21], while low field ^1^H NMR relaxometry assesses the mobility of supramolecular domains and/or water molecules via ^1^H spin-spin relaxation time (*T_2_*) in moist samples [12], [13], [22].

A major, more experimental challenge in investigation of the hypothesized cation effects is due to the mutual interdependence of cation loading and pH. In addition to the amount and composition of organic functional groups, pH is decisive for the number of available binding sites and can therefore affect the degree, type and mechanism of cation-SOM interaction. Not only the relative number of available binding sites, but also their spatial distribution will determine the relative relevance of intermolecular and intramolecular complexation [1], [8].

The objective of this study was to assess the effect of cation treatment on a peat OM with very low effective cation exchange capacity (*CEC_eff_*) and thus with low abundance of charged functional groups. The objective was to test to which extent cross-links via multivalent cations are relevant in this sample. The main focus was given to the physicochemical structure of the OM, instead of chemical composition.

A preceding study involving addition of Al^3+^, Ca^2+^ and Na^+^ to this peat had showed that contact angle (*CA*) and water binding clearly reacted on the type of cations, but stronger effect was due to temperature pre-treatment (unpublished data). The effects of cations and pH were not separated in that study and cations were exchanged only incompletely, such that the final sample still contained significant amount of the original cations.

In this study, we therefore removed initially present cations via exchange resin and the experiments were conducted under controlled pH (1.9 and 4.1, respectively). This range of pH was chosen to address realistic range of pH for peats [23]. In addition to the cations of interest (Al^3+^, Ca^2+^ and Na^+^), NaOH was added to obtain the desired pH 4.1. At this low range of pH, formation of polymeric Al^3+^ species is minimal [24], [25], and only the carboxylic groups of the OM are deprotonated, while phenolic functional groups are completely ionised only above pH 11 [25]. Such high pH is not realistic for peats [23]. We investigated effect of this cation treatment on the OM matrix properties via DSC, ^1^H-NMR-Relaxometry and ^1^H NMR wideline spectroscopy as well as on the peat-water *CA*. *CA* reflects surface changes brought about by cation binding, by orienting hydrophilic functional groups to the interior of OM matrix in the same way as other chemical changes do [18]. In order to separate the process of cation-OM interaction from that of pH-dependent cation exchange, pH was controlled either during the 24 h cation treatment phase or only 2 h before end of the cation treatment phase.

## Materials and Methods

### Materials

A sapric peat (SP) which has already been described in detail by Jaeger et al. [26] has been used for the experiments as it provides a highly complex organic matrix with 52% organic carbon (w/w). *CEC_eff_* was 123±12 mmol_c_kg^−1^, organic C content was 539±30 mg g^−1^ and pH was 2.7. The sample was air-dried, sieved (<2 mm) and equilibrated at 20°C for at least 28 days prior to use. Al(NO_3_)_3_, CaCl_2_, NaCl, NaOH, HCl, HNO_3_, BaCl_2_ and MgSO_4_ were purchased from Sigma Aldrich. All chemicals used in this study were of analytical grade.

### Methods

#### Removal of cations originally present in the peat

40 g peat was first shaken for 24 hours at a speed of 15 rpm in 900 ml of de-ionised water, with a non-selective Amberlite resin (IR 120, H^+^ form with exchange capacity of 2.3 mmol_c_g^−1^ from Merck, Darmstadt; 50 g resin per 40 g peat) packed in plastic gauzes. This resulted in pH of 1.9. One resin treated sample was isolated from the treatment solution without further treatment, to be able to distinguish the effects of cations from that of the resin treatment procedure. This sample was named “SP-H”.

#### Cation treatment

The resin packets were removed gently from the treatment solution and 100 ml of solutions of NaCl (4.9, 9.8, 19.7, 29.5 and 39.4 mmolL^−1^), CaCl_2_ (2.5, 4.92, 9.84 14.8 and 19.7 mmolL^−1^) or Al(NO_3_)_3_ (1.6, 3.3, 6.6, 9.8 and 13.1 mmolL^−1^), respectively, were added either immediately or after adjusting the pH to 4.1 using 1 M NaOH solution (5–6 ml). pH 4.1 was chosen as maximum pH in order to prevent dissolution and precipitation of Al^3+^ in the form of hydroxides which occur in the pH range of 5–8 [24], [25].

For a sketch of complete treatment procedure, please refer Figure S1 in the supporting information (SI). All steps were done in the same treatment solution, without drying the sample in between. By this procedure, we avoided potential loss of dissolved organic matter (DOM) and effects of drying.

Al^3+^, Ca^2+^ and Na^+^ were selected to represent cations of different valency. Their amount to be applied to the peat was defined with the aim to occupy a fraction of its *CEC_eff_* by the added ions. Degree of saturation of 10, 20, 40, 60 and 80% of *CEC_eff_* were anticipated. This corresponds to cation concentrations of 12.3, 24.6, 49.2, 73.8 and 98.4 mmol_c_kg^−1^, respectively.

In the experiments where pH was not adjusted prior to cation treatment, it was adjusted in the same solution, but after 24 h of cation treatment with an additional equilibration time of 2 h. The experiments were named as SP-M@1.9 and SP-M@4.1 where M corresponds to name of added cations and each sample was named by replacing M with the symbol of the cation used for treatment (e.g. SP-Al@1.9). Thus both SP-M@1.9 and SP-M@4.1 sample sets had final pH of 4.1. The two pH will be further referred as ‘cation addition pH’ (1.9 and 4.1, respectively for the two experiments) and ‘final pH’ (4.1 in both the experiments).

#### Isolation and storage of cation treated peat

Each peat-solution mixture was filtered through 0.45 micrometer filters under suction pressure. The filtrates were later analysed for cations and organic C content. The collected solid materials were dried at 25°C in a thermostat for three days and then equilibrated for at least two weeks in an atmosphere with 76% relative humidity, maintained using saturated NaCl solution, prior to any analysis. One untreated SP sample was stored in the same way and subjected to similar analysis as done for the treated samples.

#### Analysis of sample properties

Treated samples were characterized for total metal analysis, *CEC_eff_*, mobilisability of OM by water, *CA*, thermal analysis and ^1^H NMR relaxation time and ^1^H NMR wideline spectroscopy.

#### Total metal analysis

Total cation content was determined by a microwave-assisted acid digestion in a microwave digestion chamber (Microwave MarsXpress (CEM GmbH)) using reverse aqua regia (10 ml g^−1^). All extracts were analysed using an ICP-QMS instrument (Q-ICP-MS XSeries2; Thermo Fisher Scientific, Germany) to quantitatively determine the major cations – Al^3+^, Ca^2+^, Mg^2+^, Na^+^ and Fe^3+^.

#### Dispersible colloids

Aqueous extracts of the untreated peat were obtained by shaking with de-ionised water (25 ml g^−1^). In order to separate dissolved and dispersed cationic species, they were ultracentrifuged at a speed of 44000 rpm in a Sorvall WX Ultra Series WX 90 (Thermo Fisher Scientific, Germany) ultracentrifuge. The procedure allows a cut-off of particle size higher than 8 nm for inorganic colloids and 19 nm for organic colloids [27]. The particle size distribution in the supernatant and centrifugate were analyzed by dynamic light scattering using a Delsa Nano Submicron particle size analyser (Beckman Coulter, Germany). Cation content of colloidal fraction was quantified by determining total cation content of the extract before centrifugation and of the supernatant after centrifugation. The extracts and the supernatant after centrifugation were digested in acid medium (2 ml in 10 ml reverse aqua regia) in the microwave chamber (Microwave MarsXpress; CEM GmbH) and were analysed for Ca^2+^ and Fe^3+^ in an ICP-QMS (Q-ICP-MS XSeries2; Thermo Fisher Scientific, Germany).

#### Water-extractable organic carbon

The amount of organic carbon that can be released from the soil when in contact with water was determined as dissolved organic carbon (*DOC*) in aqueous solution (2 g in 50 ml water). Samples were shaken with de-ionised water for 24 h and the extracts, after removal of solid soil and filtration through 0.45 micrometer filters, were analysed in a TOC analyser (multiNC 2100S-analytik Jena, Germany) to estimate the total organic carbon content.

#### Effective Cation Exchange Capacity (CEC_eff_)

The *CEC_eff_* was estimated by the two-step BaCI_2_-MgSO_4_ method as described by Bache et al. [28], but without buffering in order to determine the exchange capacity at the sample pH. The exchangeable cations were first exchanged against 0.1 M BaCl_2_ solution (25 ml) and the barium ions were then exchanged against 0.05 M Mg^2+^ using MgSO_4_ solution (25 ml). The amount of unexchanged magnesium ions was determined by ion chromatography (Professional IC-Metrohm, Germany).

#### Contact angle

The peat-water *CA* were measured by Wilhelmy plate method (WHP) using a dynamic contact angle meter and Tensiometer (DCAT Series 21; DataPhysics), as described by Diehl et al. [18].

#### Differential Scanning Calorimetry (DSC)

Thermal analysis to determine step transition temperature was done, as described elsewhere [20], using a DSC Q1000 (TA Instruments, Germany) with refrigerated cooling system and nitrogen as purge gas. 1–3 mg sample was placed in aluminium pans and was hermetically sealed. At least three replicates per sample were measured to account for sample heterogeneity. Measurements were conducted in the temperature range between −50°C and 110°C [20]. Baseline was corrected with the TZero Technology® by TA Instruments. Data were analysed using Universal Analysis software, version 4.1 (TA Instruments). Samples were analysed again after six months in order to investigate the effect of aging.

#### ^1^H transverse relaxation time (T_2_)

Samples were moistened to water content of 20% (w/w on dry mass basis). With this method, the mobility of water molecules in the cross-linked network of the peat organic matter is assessed [12]. ^1^H transverse relaxation decay were recorded on a Bruker Minispec 7.5 NMR Relaxometer (Bruker, Germany) at a magnetic field strength of 0.176 T applying CPMG (Carr-Purcell-Meiboom-Gill) pulse sequence using the following acquisition parameters: echo time (TE) = 0.15 ms, recycle delay (RD) = 10 s, number of echoes (NE) = 1000 and number of scans (NS) = 32. Decay curves were fitted on bi-exponential equation and the component with slowest relaxation (*T2*∼2 s) was discarded from discussion because it represents the bulk water.

#### ^1^H NMR wideline spectroscopy

Samples were measured according to the procedure described in [21], using hermetically closed 4 mm NMR zirconia MAS rotors to prevent loss of water in a static (wide line) setup. The DEPTH sequence [29] was applied to suppress the ^1^H background signal of the NMR probe. Samples were measured before and directly after heating at 110°C for 30 min with a 90° pulse of 2.7 µs and a recycle delay of 0.25 s. The spectra were then fitted with the dmfit2007 program [30] by decomposing into a Lorentzian line (representing mobile protons) and a Gaussian line (representing more rigid protons). Changes of the relative ratios of these contributions are then used as indicators for the respective mobility changes upon heating. Data were acquired at two time points separated by duration of at least six months in order to assess the effect of aging on the state of water binding.

#### Molecular modeling analysis

**Figure 1:**
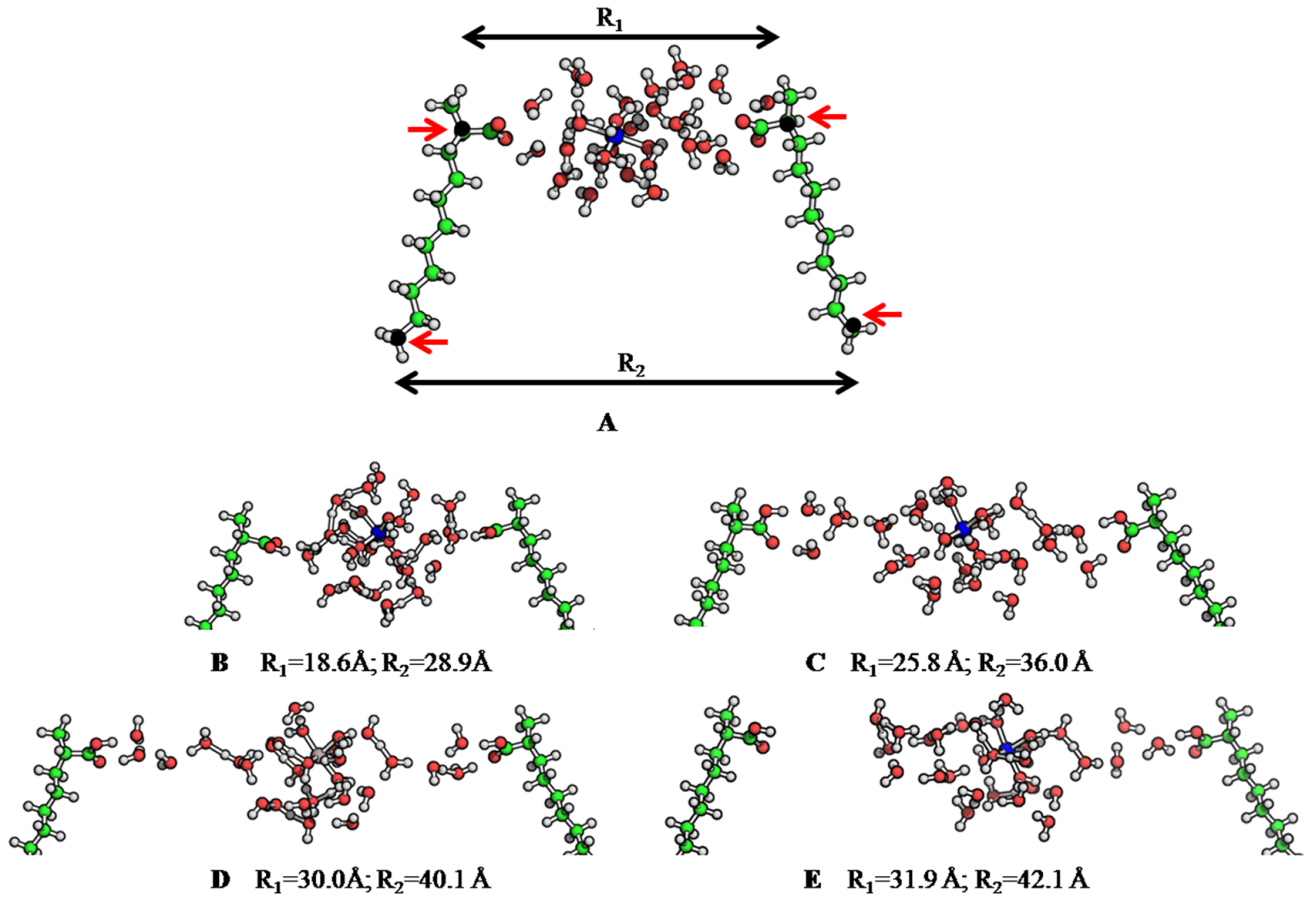
Scheme for the chosen molecular model and calculated cluster models for the hydrated aluminum cation bridging two fatty acid chains. The scheme used for modeling (A) and optimized geometries of the model cluster at different R_1_ and R_2_ - (B), (C) and (D)- are shown, respectively. The spatially fixed carbon atoms are marked by black spheres in (A). For (B), R1 = 18.6 Å; R2 = 28.9 Å, (C) R1 = 25.8 Å; R2 = 36.0 Å, (D) R1 = 30.0 Å; R2 = 40.1 Å and (E) R1 = 31.9 Å; R2 = 42.1 Å.

In order to analyze the capacity of a hydrated multivalent cation cluster to hold two polar groups together which would be otherwise non-interacting, we conducted an exemplary molecular modeling study. This type of mechanism of cross-linking humic substance units has been intensively discussed in literature (see e.g. [8] and references therein). One main question concerns the spatial contribution to the binding power of the cation. To address it, a model has been constructed based on analogous previous work [1] where a cluster of Al^3+^ surrounded by thirty water molecules is placed between two fatty acid chains positioned parallel to each other (see Figure 1A for the scheme). They were simultaneously moved away from the aluminium cluster and at each selected distance a full geometry optimization of the obtained system was performed. In this procedure the two terminal carbon atoms and the two carbon atoms linked to the carboxyl groups were kept fixed. In total, seven structures with different interchain distances R_1_ and R_2_ were generated varying between ∼19-32 Å (R_1_) and 29-42 Å (R_2_), respectively. All calculations were performed by means of density functional theory (DFT) using the PBE functional. The TZVP [31] basis set was chosen for the carbon and oxygen atoms of the carboxylate groups and for Al^+3^. For all remaining atoms the SV(P) [32] basis set was employed. The calculations were carried out with the TURBOMOLE [33] program suite.

## Results and Discussion

### Effect of Cation Treatment on General Soil Parameters

#### Effective Cation Exchange Capacity (CEC_eff_)

The untreated peat had *CEC_eff_* of 123±12 mmol_c_kg^−1^, while most treated samples revealed higher exchange capacity ranging from 160 to 240 mmol_c_kg^−1^ and SP-H had 256±23 mmol_c_kg^−1^ (see Tables S1 and S2 for values of individual SP-M@1.9 and SP-M@4.1 samples, respectively). These values are lower than CEC of organic matter in soils at least by factor of 10. CEC of SOM ranges between 1800 and 5000 mmol_c_kg^−1^ [5]. Therefore, the peat in this investigation reveals an extremely low abundance of exchange sites and the expected distance between exchange sites is considerably higher than in SOM. Assuming the very improbable case of equal distribution of functional groups within the organic matter and an OM density of 1–1.5 g cm^−3^, it leads to average distances of 3 nm between charged functional groups in the peat. It is significantly larger than expected for SOM (0.9–1.2 nm, estimated under the same assumptions as above), owing to their higher CEC. Although functional groups are expected to be distributed unevenly and to occur as hotspots in OM, this comparison shows that the probability for a functional group to have sufficiently close neighbor to form CaB is lower in the peat OM than in SOM.

**Figure 2:**
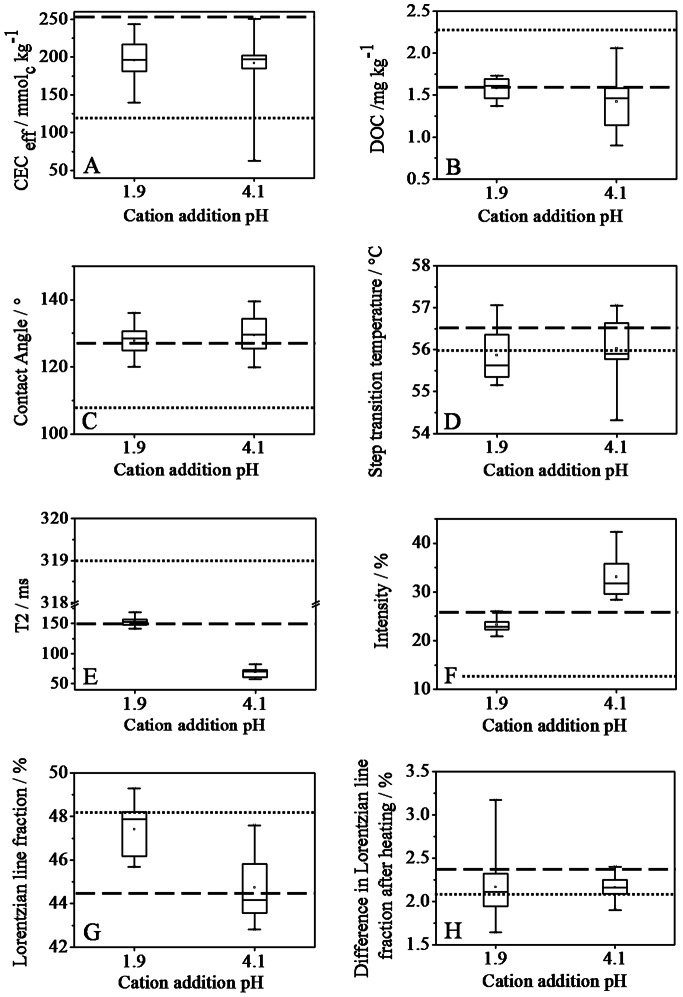
Investigated soil properties with respect to the cation addition pH. (A) *CEC_eff_*, (B) *DOC*, (C) CA, (D) T^*^, (E) T_2,fast_ and (F) contribution of fast relaxing water molecules to the total T_2_ decay, (G) contribution of Lorentzian line to the ^1^H wideline and (H) Difference in the contribution of Lorentzian component after heating event. Dotted lines and dashed lines represents the average values observed for SP and SP-H samples, respectively.

Figure 2A shows *CEC_eff_* for all treated samples in dependence of the cation addition pH and demonstrates that it is independent of cation addition pH. Thus, neither cation treatment (see Tables S1 and S2) nor cation addition pH affected *CEC_eff_*, but the final pH (4.1in treated samples and 2.7 in untreated sample). This result can be explained with the higher degree of deprotonation of acidic functional groups at higher pH. This demonstrates that (1) 2 h of equilibration are sufficient to obtain a protonation/deprotonation equilibrium and (2) cation treatment did not affect *CEC_eff_*.

#### Water-extractable organic carbon

The water-extractable organic carbon, denoted here as *DOC* of the untreated and resin-treated samples were found to be 2.3±0.1 mg kg^−1^ and 1.6±0.1 mg kg^−1^, respectively. The values for treated samples after drying and equilibration, ranged between 1.0 and 2.2 mgkg^−1^, without any significant dependence on the type or amount of incorporated cation or on the treatment pH (see Figure 2B and Tables S1 and S2 for more details). Organic carbon content of the resin-treatment solution was 0.8±0.1 mg kg^−1^, showing that mass balance exists and hence no water soluble organic C was lost by sorption to the resin.

Lower *DOC* in cation treated samples than in untreated peat contradicts the general expectation that OC solubility increases with increasing pH. One explanation could be that OM was removed during cation treatment. This is supported by organic C content of the treatment solutions indicating removal of 0.2–1.3 mg kg^−1^ organic C, varying with treatment, but independent of cation type or concentration. This further suggests that only a small fraction (less than 1%) of total organic C was lost during the treatment.

### Cation Content after Treatment

**Figure 3:**
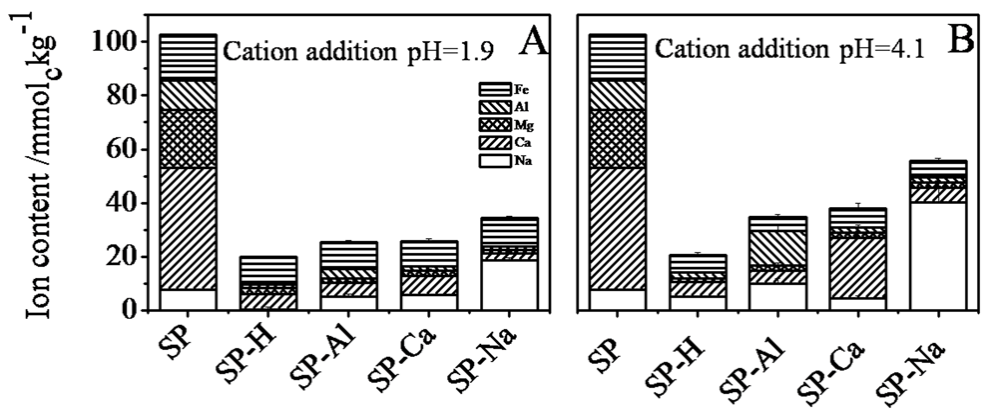
Effect of sample treatment by exchange resin followed by addition of specific cations on the major cation content with respect to different charging pH, shown for highest cation loading. Also the values for untreated (SP) and exchange resin-treated samples (SP-H) are shown.

Figure 3 shows the cation composition of peat – without any treatment, after removal of cations and after addition of cations. Data for samples with highest cation loading are shown, exemplarily. The untreated peat contained mainly calcium (45.3±0.5 mmol_c_kg^−**1**^), magnesium (21.6±0.3 mmol_c_kg^−**1**^), iron (16.9±0.1 mmol_c_kg^−**1**^), aluminium (11.1±3.2 mmol_c_kg^−**1**^), and sodium (7.8±0.3 mmol_c_kg^−**1**^). Cation removal by exchange resin (sample SP-H in Figure 3A) resulted in removal of 84% of initially present cations, retaining 5.7±0.4 mmol_c_kg^−**1**^ calcium, 2.1±0.2 mmol_c_kg^−**1**^ magnesium, 6.7±0.1 mmol_c_kg^−**1**^ iron, 1.3±0.4 mmol_c_kg^−**1**^ aluminium and 0.21±0.03 mmol_c_kg^−**1**^ sodium. As expected, higher sodium content (5.0±1.0 mmol_c_kg^−**1**^) was observed in exchange resin-treated samples of the experiment where pH was later adjusted to 4.1 using NaOH. Iron and calcium were comparably less affected by treatment with exchange resin: 40% and 15% of their initial amounts were, respectively, retained. The incomplete removal of these cations during resin treatment indicates that they are only partly bound via cation exchange, and will be discussed further below.

Cation addition after resin treatment resulted only in partial uptake satisfying 25% of the initially present cationic charge equivalence at cation addition pH 1.9 and 34% at cation addition pH 4.1. In both cases, uptake was significantly higher for SP-Na samples than for SP-Al and SP-Ca. Furthermore, cation uptake was affected only by pH during the 24 h cation treatment phase. Comparing the cation uptake in terms of total charge equivalence, addition at pH 4.1 (12.8±2.3 mmol_c_kg^−**1**^, 22.5±0.7 mmol_c_kg^−**1**^, 40.3±10.4 mmol_c_kg^−**1**^ for SP-Al, SP-Ca and SP-Na, respectively) was considerably more effective than at pH 1.9 (3.3±0.1 mmol_c_kg^−**1**^, 7.2±0.1 mmol_c_kg^−**1**^ and 18.7±1.0 mmol_c_kg^−**1**^ for SP-Al, SP-Ca and SP-Na, respectively, Figure 3). This can be explained by the pH dependence of deprotonation status of the OM, as revealed by pH titration curve of the original peat (see supporting information, Figure S2). The titration curve shows that significant buffering against addition of NaOH starts only above pH 11 and a large slope (0.3 kg cmol_c_^−1^) in the low pH range till pH 4 is indicative for strong dissociation of acidic functional groups. Further, the pH varied linearly in the range 4–11 without change in slope which is unexpected in comparison with general SOM acidity [24], [25]. This may suggest an overlay of buffering effects due to strongly bound Al, dissociation of aliphatic and aromatic carboxylic groups till pH 8 and dissociation of phenolic groups which become prominent above pH 8.5. Polysubstitution at aromatic ring structures by –COOH and –OH might have caused further broadening of buffering response [25].

Nevertheless, pH adjustment to 4.1, carried out only 24 h after cation addition and during a 2 h equilibration phase still resulted in a lower cation uptake (25% of initial CEC) than for cation addition at pH 4.1 (34% of initial CEC). This indicates that the 2 h contact time after pH adjustment was not enough for cations to react on the increase in number of exchange sites.

Summarizing, the actual cation uptake depended on the cation addition pH, in contrast to the *CEC_eff_* which depended on the final pH. Furthermore, cation addition resulted in significantly lower cation uptake in relation to cation content of the original peat, even though the final pH was higher than pH of the original sample. Also, the relatively short equilibration time of 2 h after pH adjustment to 4.1 in respective experiments, was probably not enough to break compactness of the system, whereby only a few exchange sites are opened for newly added cations.

Thus, cation uptake was controlled not only by the exchange capacity of peat, but some kinetic limitation must have impeded cations from occupying the exchange sites. The resin treatment and/or the accompanied pH reduction may have changed the accessibility of cation exchange sites. Protonation of the system by the resin reduces the negative charge density, allowing closer approach of functional groups in those regions where repulsive forces had caused larger distances at higher pH. This can increase the degree of hydrogen bonding in the OM, which could result in a highly cross-linked hydrogen bond network where accessibility of some exchange sites gets reduced. This explanation indirectly suggests that the acidic functional groups are not evenly distributed in the OM.

The lower efficiency of Al^3+^ and Ca^2+^ than Na^+^, to enter the peat, could be due to the difference in hydrated ionic radii (480 pm, 412 pm, 358 pm for Al^3+^_aq_, Ca^2+^_aq_, and Na^+^_aq_, respectively [34]), resulting in lower diffusion coefficients in hydrated organic matter. This also would indicate that kinetic effects restrict the occupation of cation exchange sites.

### Resistance of Iron and Calcium against Exchange Resin-treatment

As discussed above, iron and calcium content of treated samples were not affected significantly by exchange resin treatment although these two ions have been detected in the supernatant solution during exchange resin-treatment (data not shown). Moreover, a higher degree in removal of calcium than iron suggests that the cation removal may have partly occurred in a selective way or that iron and calcium occur in different binding states in the organic matter. The observation that no significant amount of carbon was lost during resin treatment (chapter 3.1.2) suggests that DOM did not significantly sorb to the exchange resin and hence the possibility of modified functioning of resin can be neglected. Thus, larger part of Fe than of Ca is more stable towards cation exchange.

Both cations may exist in this peat as stable but mobilisable species; for example, as organic or inorganic colloids. This hypothesis was tested by centrifugation-induced sedimentation of dispersed species in aqueous extracts followed by particle size measurement. In the supernatant, particles were not detected whereas the centrifugate revealed particles of size 400±20 nm (data not shown). Elemental analysis of the centrifugate showed calcium and iron as major constituents (see Figure S3). This supports the assumption that iron and calcium were mobilised as inorganic or organic colloidal particles. Furthermore, iron may occur in part as polymeric species, which would explain the higher resistance against cation exchange than Ca. Binding state and stability of these colloids were not affected by the pH in the investigated range, and the cations are not exchangeable. The nature of these colloids needs to be investigated in a separate study.

### Cation Sorption Isotherms

**Figure 4:**
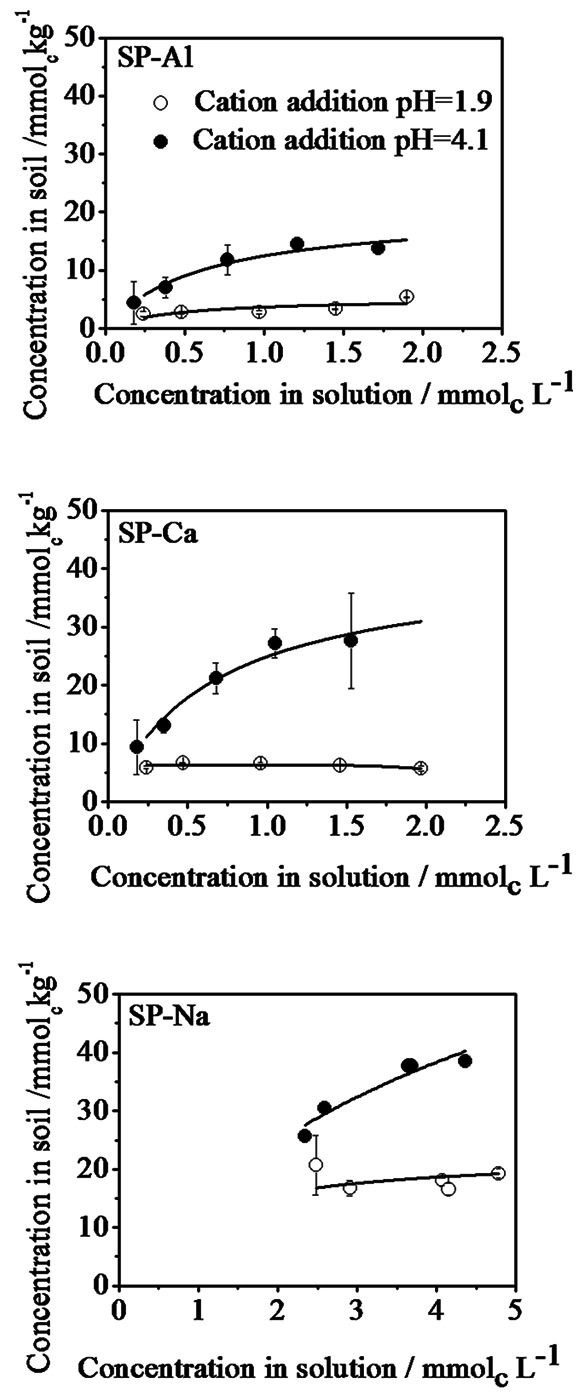
A comparison of cation uptake with respect to the amounts remained in treatment solution in aluminium (SP-Al), calcium (SP-Ca) and sodium (SP-Na) treated samples at different cation addition pH.

The dependence of efficiency of cation uptake on amount of added cations is shown in sorption isotherms in Figure 4. The higher uptake of the respective cation at higher cation addition pH demonstrated by Figure 3 is even clearer in the sorption isotherm. Shapes of the isotherms were non-linear, but similar for all conditions. The sorbed amount increased with equilibrium solution concentration and appeared to approach a limit value, for higher equilibrium solution concentrations, which depends on cation type and cation addition pH. Only for Na^+^, the limit value was not reached under the conditions applied in the current study. Sorption isotherms for cation addition at pH 1.9 resulted in lower limit values and the final value were already reached at low equilibrium concentrations.

Sorption curves were analysed by fitting Langmuir sorption equation (1) to the data using Levenberg-Marquardt algorithm using Origin 7.5 software (from OriginLab).
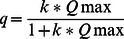
(1)where *q* is the concentration of the analyte sorbed to the solid phase at respective concentration in solution, *k* is the Langmuir constant which reflects sorption affinity of the sorbent-sorbate system under consideration and *Qmax* represents maximum sorbable amount, beyond which sorbed amount will not increase even with increase in solution concentration.

Sorption occurring at pH 4.1 was well described by Langmuir function (R^2^≥0.96) whereas fit of sorption data at pH 1.9 resulted in larger scattering (R^2^ = 0.7). Fitting with Langmuir model resulted in higher quality than with Freundlich and linear sorption isotherms. Latter resulted in significant systematic deviation (data not shown). Good fit to the Langmuir isotherm supports the assumption that cation sorption reached its maximum under the experimental conditions of this study. It may further invoke that sorption involves specific binding at sufficiently separated functional groups [35]. But on contrary to Langmuir sorption on surfaces [36], it does not necessarily indicate homogeneity in distribution of functional groups.

The curve fitting parameters are shown in Table 1. The Langmuir coefficient *k* varied between 0.6 and 3.9 and is comparable for both pH values and for all three cations. *Qmax* is considerably lower for SP-M@1.9 than that for SP-M@4.1 samples and still lower than *CEC_eff_* determined in exactly the same samples in both cases (see Tables S1 and S2). Furthermore, in contrast to expectation, *Qmax* depended on cation type in the order: Al^3+^<Ca^2+^<<Na^+^, in both the experiments, indicating a decreasing exchange efficiency with increasing cation valency.

**Table 1:** Langmuir fit parameters of sorption curves.

Experiment	Added cation, M	*Qmax*/mmol_c_kg^−1^	*K* /mmol_c_^−1^kg	R^2^
	Al	4.01±1.93	0.91±0.89	0.69
SP-M@1.9	Ca	0.91±1.37	3.91±2.79	0.70
	Na	23.01±3.25	1.01±0.29	0.68
	Al	20.23±5.26	0.99±0.53	0.98
SP-M@4.1	Ca	47.15±16.76	0.64±0.38	0.97
	Na	91.49±13.37	0.65±0.09	0.98

The comparable *k* values irrespective of cation type and cation addition pH suggest comparable affinity of all three cations to binding sites, regardless of charge. This is in contrast to the general observation in soils that cation exchange affinity depends on the electrostatic interaction force between exchange groups and cation and therefore increases with increasing cation valency.

Cation addition pH mainly affected the maximum exchange capacity. However, the unexpected difference in *Qmax* for the two experiments, for all three cation types, again shows the importance of cation addition pH in determining the available exchange capacity. Cation exchange would result in similar *Qmax* for all the three cations at a particular pH since the number of available exchange sites is equal. The observed dependence, therefore, suggests that not all cation exchange sites are accessible by all cations, and the accessibility depends on the cation valency and on the experimental conditions during cation exchange. Considering SP-Al and SP-Ca samples in Figure 3, it can be seen that the rest of the available exchange sites at a specific pH, after binding of Al^3+^/Ca^2+^ are occupied by Na^+^. Thus exchange sites that are not accessible by Al^3+^ or Ca^2+^ were occupied by Na^+^. This might be because of the higher abundance of Na^+^ (125–150 mmol_c_kg^−1^) due to pH adjustment, which was 10–80 times higher than the amount of Al^3+^/Ca^2+^ added in various experiments. This, however, cannot explain the lower *Qmax* for cation addition at pH 1.9, because Na^+^ were not present during the 24 h equilibration time. Another explanation could be a restriction of accessibility of exchange sites in the hydrated organic matter, with respect to the size of approaching cation. Na^+^, being the smallest in hydrated state [34], reaches the most cation exchange sites whereas Al^3+^ and Ca^2+^ cannot access all sites due to their larger size. Our results thus suggest that part of the exchange sites are separated by small nanopores too small for Ca^2+^_aq_ or Al^3+^_aq_ to pass through. Additional argument for low uptake of Al^3+^ could be based on the fact that it can form polymeric ions which are too big to occupy the voids between OM molecules or to penetrate into the nanopores. However, only a very small fraction (<10 ppm) of total Al^3+^ contributes to such species, at the low pH range of this study [37]. The low concentration of added solution further lowers significance of this effect. Moreover, a pronounced effect of polymeric Al species would have caused higher uptake of Al^3+^ at pH 1.9 than at pH 4.1, because polymeric and complex hydroxyl species are more prominent at pH 4.1 [37]. But our observation that cation uptake was higher at pH 4.1 than at pH 1.9 contradicts this and suggests further that pH-dependant speciation of Al^3+^ did not affect cation uptake, significantly.

The observation that the *CEC_eff_* was affected only by the final pH (Figure 2A) regardless of cation type or cation addition pH suggests that the procedure to determine *CEC_eff_* opens these nanopores, so that the higher concentration of Ba^2+^ than Na^+^, Ca^2+^, or Al^3+^, respectively, leads to a more effective exchange process. However, the observation that cation sorption approaches *Qmax* significantly lower than *CEC_eff_*, contradicts this assumption and suggests that the high concentration of Ba^2+^ enables opening of the nanopores. Another possible explanation why Na^+^ reveals the highest uptake could be that multivalent cations need to bind with more than one organic functional groups to satisfy their complete charge. This will lead to intra- or intermolecular cross-linking, where neighbouring exchange sites are available in sufficient proximity [1], [8]. Due to the extremely low cation exchange capacity and based on the hypothesis that distances between many charged functional groups and their closest neighbours are much larger than in regular SOM, these results suggest that many functional groups cannot be bridged by hydrated multivalent cations in outer sphere complexes (<<1 nm [1]) or by WaMB-CaB associations (2 nm for up to 10 water molecules [1], [11]). To be able to estimate distances which could be bridged by CaB-WaMB associations with more than 10 water molecules, we conducted an additional set of molecular calculations.

### Modeling CaB-WaMB Associations to Bridge Large Distances of Functional Groups

The optimized structures obtained for selected interchain distance are displayed in Figure 1. It is important to note that the geometry optimization performed at the smallest distance (R_1_ = 18.6 Å) immediately led to proton transfer from two water molecules to the COO^−^ groups forming OH and COOH groups. This arrangement was used in all following calculations. Figure 1B shows the coordination shells of aluminium cation and the hydrogen bonded water network connecting the two carboxylic groups. On increasing R_1_ up to ∼25.8 Å, the cation bridge remains well established since the hydrated aluminium cation cluster is directly connected via hydrogen bonds to the carboxyl groups (Figure 1C). Beginning with the distance R_1_ about 28 Å this connection starts to break (Figure 1D). At R_1_ = 31.9 Å one of the carboxyl groups is already completely isolated and the other carboxyl group is only weakly connected with the aluminium cluster (Figure 1E).

The interchain distances selected in these calculations depend, of course, on the size of the water cluster around aluminium cation. The radius of the water sphere around the cation in structures of Figure 1 is ∼5.5 Å. It takes a displacement of about 4.7 Å from each carboxylic site to significantly weaken the hydrogen bond connection to the carboxylic groups. This distance corresponds approximately to two water molecules bridging the carboxylic groups and the aluminium/water cluster. From these results we can conclude that a displacement of ∼5 Å is required also for other sizes of the aluminium hydration shell.

Consequence of these modelling results is that many functional groups cannot be bridged readily if their distance is too large. Increasing number of water molecules in the modelling approach will lead to the potential to bridge distances above 5 Å. However, formation of such large water clusters requires the presence of significant amount of water molecules at the correct location. It is most probable that the larger the cluster size required to bridge two functional groups, the lower the probability of formation right in the beginning. The large clusters may, however, be formed successively by aging processes.

### Soil Surface Properties and Physicochemical Characteristics

#### Contact angle

The peat-water *CA* of the untreated air-dried peat was 109±2°. Cation treatment increased the contact angle to 120–140°, independent of cation type and amount (Tables S1 and S2). Also cation addition pH did not cause considerable differences, as seen from Figure 2C. Thus the cation treatment increased hydrophobicity irrespective of cation type. The current data indicates that the low cation uptake is not significant enough to be reflected in *CA*. Furthermore, sensitivity of *CA* towards changes in surface hydrophobicity is low for such high values [18], [38].

#### DSC Step Transition Temperature (T^*^)

The DSC step transition temperature attributed to WaMB transitions [12], [20] ranged between 54°C and 58°C in all treated samples including SP-H (T^*^ = 56.5±0.5°C) whereas T^*^ for untreated peat was 56.0±0.3°C. It did not reveal significant dependence on the type and amount of cations (Tables S1 and S2) or on the cation addition pH (Figure 2D). Thus, the cation treatment did not affect the OM matrix rigidity in a measurable manner and the functional groups of the peat OM did not undergo significant cross-linking, which is against the general expectations [4], [8], [39], [40]. This observation can be explained considering the extremely low *CEC_eff_* of the peat and the low abundance of charged functional groups under the assumption that only low number of functional groups are close enough to form cross-links in the peat OM.

#### ^1^H NMR transverse relaxation time (T_2_)

Figure 2E shows results of the transverse proton relaxation time analysis. The relaxation decay was dominated by a slowly relaxing component (*T_2,slow_* = 2000±300 ms) corresponding to relaxation of liquid-like water and a fast relaxing component (*T_2,fast_* = 60 ms to 335 ms for the complete set of samples) corresponding to water molecules of restricted mobility which are in contact with the OM matrix, forming surface water and/or pore water [26]. A higher relaxation time can indicate either larger pore diameters or higher mobility of water molecules [26].

*T_2,fast_* was 319±16 ms for untreated peat, and it was considerably reduced by cation treatment to 153±7 ms and 70±7 ms for SP-M@1.9 and SP-M@4.1, respectively (Figure 2E). Within each cation addition pH, cation type and amount did not affect *T_2,fast_* (Tables S1 and S2). *T_2,fast_* revealed by SP-H (150±17 ms) was similar to the SP-M@1.9 samples. Figure 2F shows a box plot of relative contribution of the fast relaxing component to the net relaxation. This fraction was 23±4% of the total water for SP-M@1.9 samples, 32±10% for SP-M@4.1 samples, 12±6% in untreated peat and 26±3% in SP-H. Both cation treatments thus resulted in a higher extent of water interaction with peat, with higher percentage for cation addition at pH 4.1 than at pH 1.9. The peat thus was able to interact with more water molecules when cations were added at higher pH. Due to the shorter relaxation time, those water molecules were also stronger restricted in mobility than for cation addition at pH 1.9. This can be due to the higher amount of cations in SP-M@4.1 samples than in SP-M@1.9 samples.

On contrary, the general reduction in proton relaxation time and increase in amount of immobilized water upon cation treatment indicate reduction in molecular mobility of water upon the treatment procedure although the net cation concentration decreased. This could be due to the increase in final pH from 2.7 (untreated) to 4.1 (treated), resulting in a higher concentration of charged functional groups. It would require more hydration water and eventually also more WaMB. Still, increase in strength of WaMB was not observed in T^*^, which again demonstrates that at extremely low CEC of the peat under investigation, cross-linking is a less significant process affecting matrix properties than for SOM. However, the explanation based on the final sample pH is contradicted by the results shown by SP-H, which has the lowest final pH (1.9), but both the *T_2,fast_* and relative amount of corresponding water molecules lied very close to the values revealed by SP-M@1.9 samples. But this similarity cannot be discussed in the current state because also the SP-M@4.1samples, that revealed lower *T_2,fast_*_,_ had passed the state of pH 1.9 during treatment procedure. The lower proton mobility in SP-H sample than in the untreated peat could be due to a higher matrix rigidity via H-bonding since the sample would be rich in protons after the resin treatment. Probably this effect was too small to be observed in T^*^. Altogether, it can be seen that binding of water molecules in this peat is controlled by several overbalancing factors such as pH, amount of cations and matrix rigidity, which cannot be resolved further in the current study.

#### ^1^H wideline NMR

The Lorentzian line before the heating event represented 46–50% of the proton wideline signal for cation addition pH 1.9, while this percentage was slightly higher for the cation addition pH 4.1 (43–48%; Figure 2G). Similar to the trend revealed by *T_2,fast_,* mobile water fraction for untreated peat (48.0±0.2%) was higher than all the treated samples (with one exception) and that for resin treated sample (44.2±0.2%) was comparable to the SP-M@1.9 samples. The heating event led to an increase of the Lorentzian fraction by 2.2±0.4% and 2.2±0.1% for cation addition at pH 1.9 and 4.1, respectively. The values were 2.1±0.2% and 2.4±0.2% for untreated and resin-treated samples. Thus, mobile water was significantly lower in relevance for the higher cation addition pH, and water mobilisable by the short heating event did not differ significantly between different cation treatments. The less mobile water in SP-M@4.1 could be because of that more water molecules get immobilized in hydration shells as cation content is higher than in SP-M@1.9.

In contrast to the cation addition pH, cation concentration and cation type had no significant effect on mobile water (Tables S1 and S2). However, the mobilisable component decreased significantly with increasing cation valency (P<0.15 and P<0.06 for pH 1.9 and 4.1, respectively; see Figure S4), indicating decrease in WaMB water [21] with increasing cation valency.

### Aging Effects on OM Rigidity and Water Mobility

**Figure 5:**
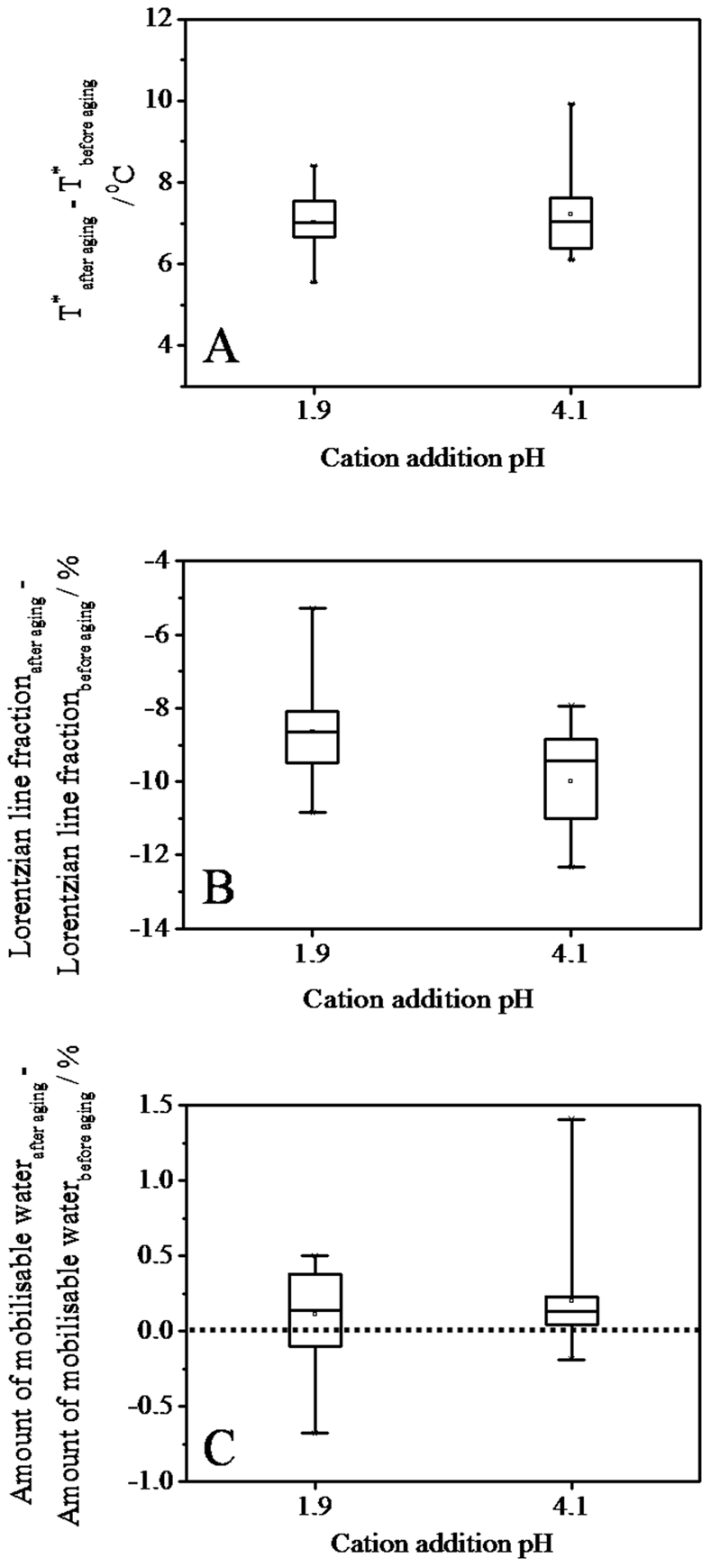
Aging effects on WaMB transition temperature (A), Lorentzian line fraction (B) and on the amount of mobilisable water (C), expressed by the difference of the respective parameters after and before aging. Positive values indicate an increase, and negative values indicate a decrease in the parameter, respectively. Dotted line along zero shown in (C) distinguishes the positive and negative effects.

Sample storage for six months under defined conditions (T = 20°C, RH = 76%) resulted in significant changes of matrix rigidity, mobile and mobilisable water. T^*^ increased significantly by 5–10°C and 4°C for the treated and untreated peat, respectively (Figure 5A). The increase was independent of cation type and loading (data not shown). The ^1^H wideline Lorentzian line intensity, indicating mobile water, decreased by 6–9% for pH 1.9 and by 8–12% for pH 4.1 samples, respectively (Figure 5B). The decrease was strongest for Na^+^-treated samples and weakest for Al^3+^-treated samples (Figure S5), but did not vary with cation loading within each set of cation treatment. Percentage of mobilisable water increased for cation addition at pH 4.1 in almost all samples (except the SP-Na sample with lowest loading), while for pH 1.9, mobilisable water of all Na^+^- treated samples increased, whereas it even decreased in some Ca^2+^- and Al^3+^- treated samples (3 out of 10 cases) (see Figure 5C and Figure S6 for details). This underlines again that the Na samples are subjected to strongest aging.

Aging for six months thus increased matrix rigidity and WaMB water and reduced the mobile water component. The aging effect appeared to be cation dependent, with strongest aging effects for the lowest valency and lowest aging effects for the highest cation valency. The aging process might have induced rearrangement of cations and water molecules within the OM matrix with the consequence of increasing amount and stability of WaMB with aging time [20], [41]. The stronger increase in matrix rigidity in cation treated samples than in the untreated sample suggests that the treatment procedure can trigger further rearrangement in the OM.

### Conclusions

Physicochemical properties of the peat investigated in this study did not reveal significant response to cation treatment, which contradicts the idea that multivalent cations cross-link molecular segments of organic matter [4], [8], [39], [40]. Thus, at least in the investigated peat, cross-linking via multivalent cations is not a relevant process. The unexpected effects can be attributed to the low cation exchange capacity of the peat. The resulting low abundance of charged functional groups suggests that the majority of functional groups have distances too large to be bridged by cations of by CaB-WaMB associations.

Cation sorption is significantly slower than deprotonation of the functional groups and follows Langmuir-like isotherms, where *Qmax* decreases with increasing cation valency. This suggests a certain uneven distribution of functional groups. Even when assuming that the exchange sites are not equally distributed, a complete accumulation at certain hotspots will be hindered by steric restrictions given from the molecular and supramolecular arrangement as demonstrated in molecular modeling studies [4], [42].

Complementarily, this observation can be explained by large average distances between functional groups, which prevent binding of a cation to more than one functional groups even if it would require more functional groups for charge saturation: Al^3+^ would require even three functional groups in sufficient proximity. If these are not available, an excess positive charge will remain, rendering the exchange process at this specific location less favorable, as counter ions have to be taken up in order to ensure charge neutrality. In contrast, Na^+^ can attach to any functional group without requiring additional exchange sites. Thus, monovalent cations may find more cation exchange places than the trivalent Al^3+^ in this peat due to its low exchange capacity.

The low potential of the peat for cross-linking via cations is furthermore shown by the low amount of WaMB water determined from the NMR wideline analysis and by the only weak dependence of WaMB amount (NMR wideline), matrix rigidity (T^*^) and water mobility (*T_2_*) on the cation type and cation loading, and it is further supported by the molecular modelling findings.

Sample aging, however, resulted in clear increase in WaMB water in most cases, in increase in T^*^ and in decrease in mobile (Lorentzian) water. This indicates an aging-induced increase in matrix rigidity and an increasing extent of water binding in the course of aging, which is most probably due to the slow rearrangement of OM molecular segments, cations and water molecules, increasing the degree and stability of WaMB and therefore the degree of cross-linking in the peat organic matter despite its low functional group density.

## Supporting Information

Figure S1 **Schematic diagram describing the experimental procedure adopted for treatment of peat with exchange resin and with different cations.**
(PDF)

Figure S2 **Titration curve of the original peat.** Aqueous suspension of peat was titrated against 0.1 M NaOH with time interval of 90 minutes between each titration step. This interval was selected to record stable pH.
(PDF)

Figure S3 **Cation composition of colloidal particles deposited after ultracentrifugation of aqueous extract of untreated peat.**
(PDF)

Figure S4 **Amount of mobilisable water in treated peat with respect to the type of loaded cations, after cation treatment at pH 1.9 A) and at 4.1 B) before aging**
(PDF)

Figure S5 **Lorentzian line fraction of 1H wideline (amount of mobile water) after aging, shown with respect to cation types for SP-M@1.9 (A) and for SP-M@4.1(B).** Lorentzian line fraction for all the treated samples before (t0) and after (t1) aging are shown in (C).
(PDF)

Figure S6 **Change in amount of mobilisable water after aging for cation addition at pH 1.9 (A) and at 4.1 ((B), with respect to the loaded cation type.** Dashed line along zero distinguishes the increase and decrease in amount of mobile water after aging.
(PDF)

Table S1 **All the investigated parameters for untreated (SP), resin-treated (SP-H) and cation treated samples with different cations and cation concentration where cation treatment was carried out at pH 1.9.**
(PDF)

Table S2 **All the investigated parameters for cation treated samples with different cations and cation concentration where cation treatment was carried out at pH 4.1.**
(PDF)
