# Supplementary material for: Restructuring of a Peat in Interaction with Multivalent Cations: Effect of Cation Type and Aging Time
Source: PLoS One. 2013 Jun 4;8(6):e65359. doi: 10.1371/journal.pone.0065359 (PMC3672098; doi:10.1371/journal.pone.0065359)
Supplement: Figure S1 — Schematic diagram describing the experimental procedure adopted for treatment of peat with exchange resin and with different cations. (PDF) [file pone.0065359.s001.pdf]

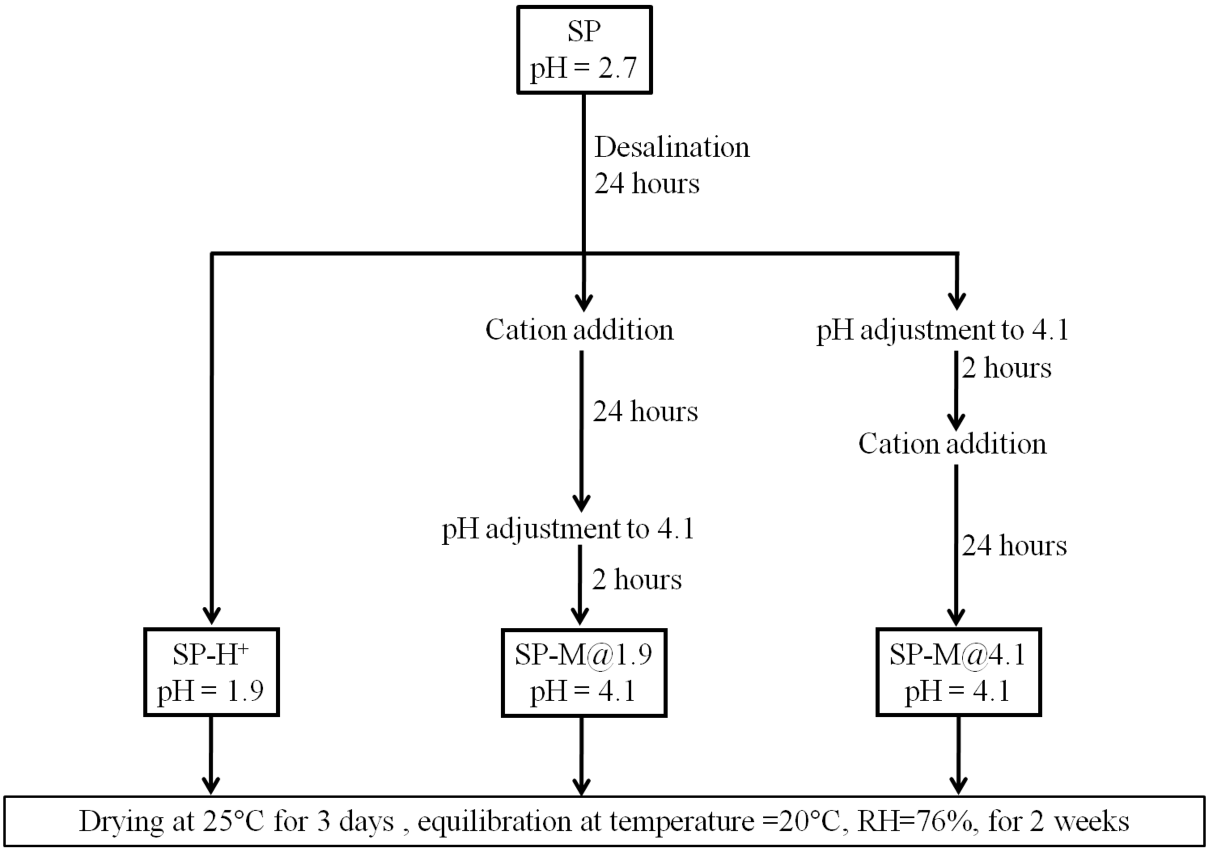

Figure S1. Schematic diagram describing the experimental procedure adopted for treatment of peat with exchange resin and with different cations.
