# Supplementary material for: Restructuring of a Peat in Interaction with Multivalent Cations: Effect of Cation Type and Aging Time
Source: PLoS One. 2013 Jun 4;8(6):e65359. doi: 10.1371/journal.pone.0065359 (PMC3672098; doi:10.1371/journal.pone.0065359)
Supplement: Figure S2 — Titration curve of the original peat. Aqueous suspension of peat was titrated against 0.1 M NaOH with time interval of 90 minutes between each titration step. This interval was selected to record stable pH. (PDF) [file pone.0065359.s002.pdf]

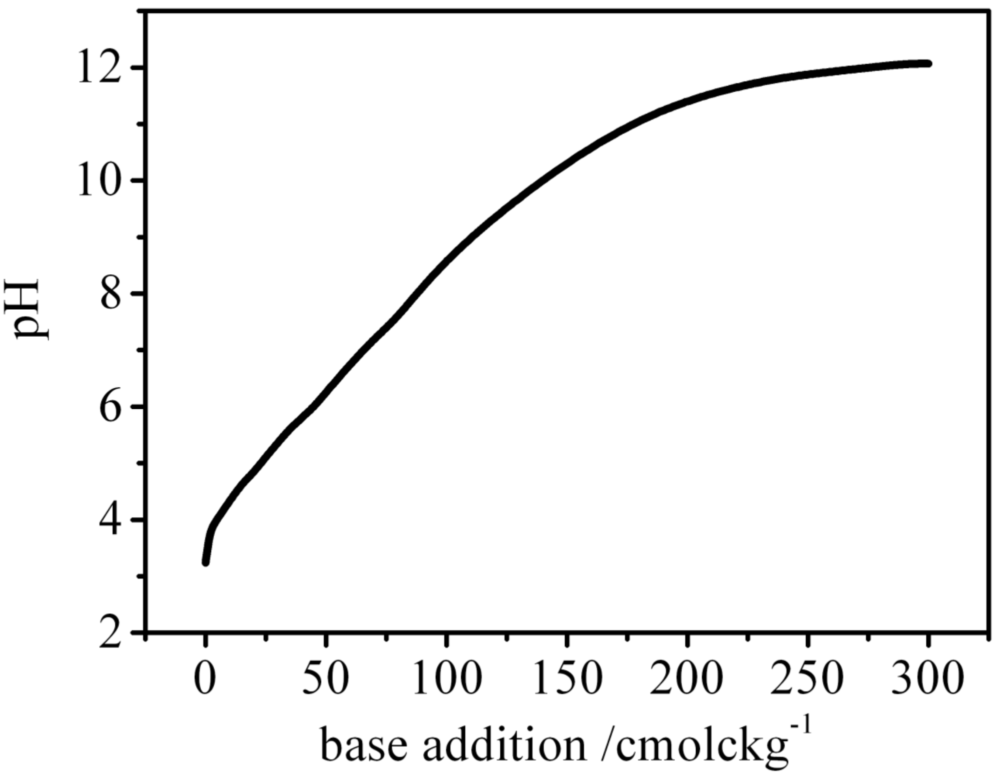

Figure S2. Titration curve of the original peat. Aqueous suspension of peat was titrated against 0.1 M NaOH with time interval of 90 minutes between each titration step. This interval was selected to record stable pH.
