# Supplementary material for: Restructuring of a Peat in Interaction with Multivalent Cations: Effect of Cation Type and Aging Time
Source: PLoS One. 2013 Jun 4;8(6):e65359. doi: 10.1371/journal.pone.0065359 (PMC3672098; doi:10.1371/journal.pone.0065359)
Supplement: Figure S3 — Cation composition of colloidal particles deposited after ultracentrifugation of aqueous extract of untreated peat. (PDF) [file pone.0065359.s003.pdf]

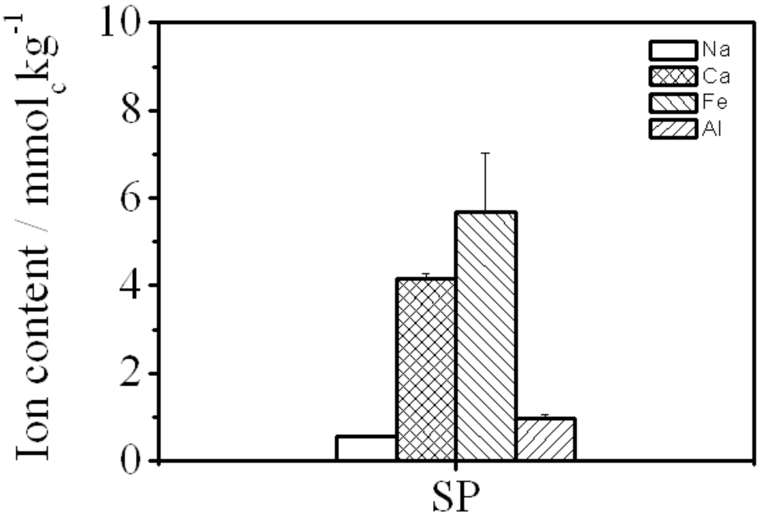

Figure S3. Cation composition of colloidal particles deposited after ultracentrifugation of aqueous extract of untreated peat.
