# Supplementary material for: Restructuring of a Peat in Interaction with Multivalent Cations: Effect of Cation Type and Aging Time
Source: PLoS One. 2013 Jun 4;8(6):e65359. doi: 10.1371/journal.pone.0065359 (PMC3672098; doi:10.1371/journal.pone.0065359)
Supplement: Figure S4 — Amount of mobilisable water in treated peat with respect to the type of loaded cations, after cation treatment at pH 1.9 A) and at 4.1 B) before aging (PDF) [file pone.0065359.s004.pdf]

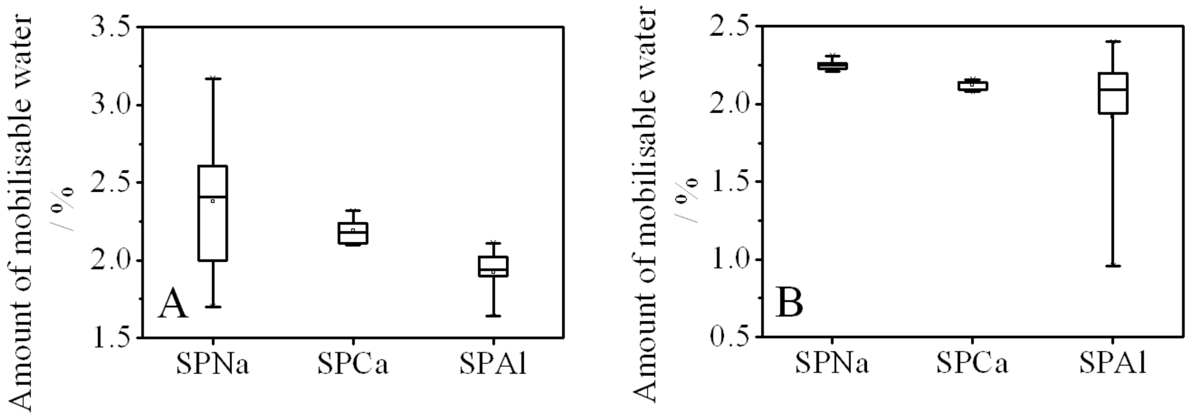

Figure S4. Amount of mobilisable water in treated peat with respect to the type of loaded cations, after cation treatment at pH 1.9 A) and at 4.1 B) before aging.
