# Supplementary material for: Restructuring of a Peat in Interaction with Multivalent Cations: Effect of Cation Type and Aging Time
Source: PLoS One. 2013 Jun 4;8(6):e65359. doi: 10.1371/journal.pone.0065359 (PMC3672098; doi:10.1371/journal.pone.0065359)
Supplement: Figure S5 — Lorentzian line fraction of 1H wideline (amount of mobile water) after aging, shown with respect to cation types for SP-M@1.9 (A) and for SP-M@4.1(B). Lorentzian line fraction for all the treated samples before (t0) and after (t1) aging are shown in (C). (PDF) [file pone.0065359.s005.pdf]

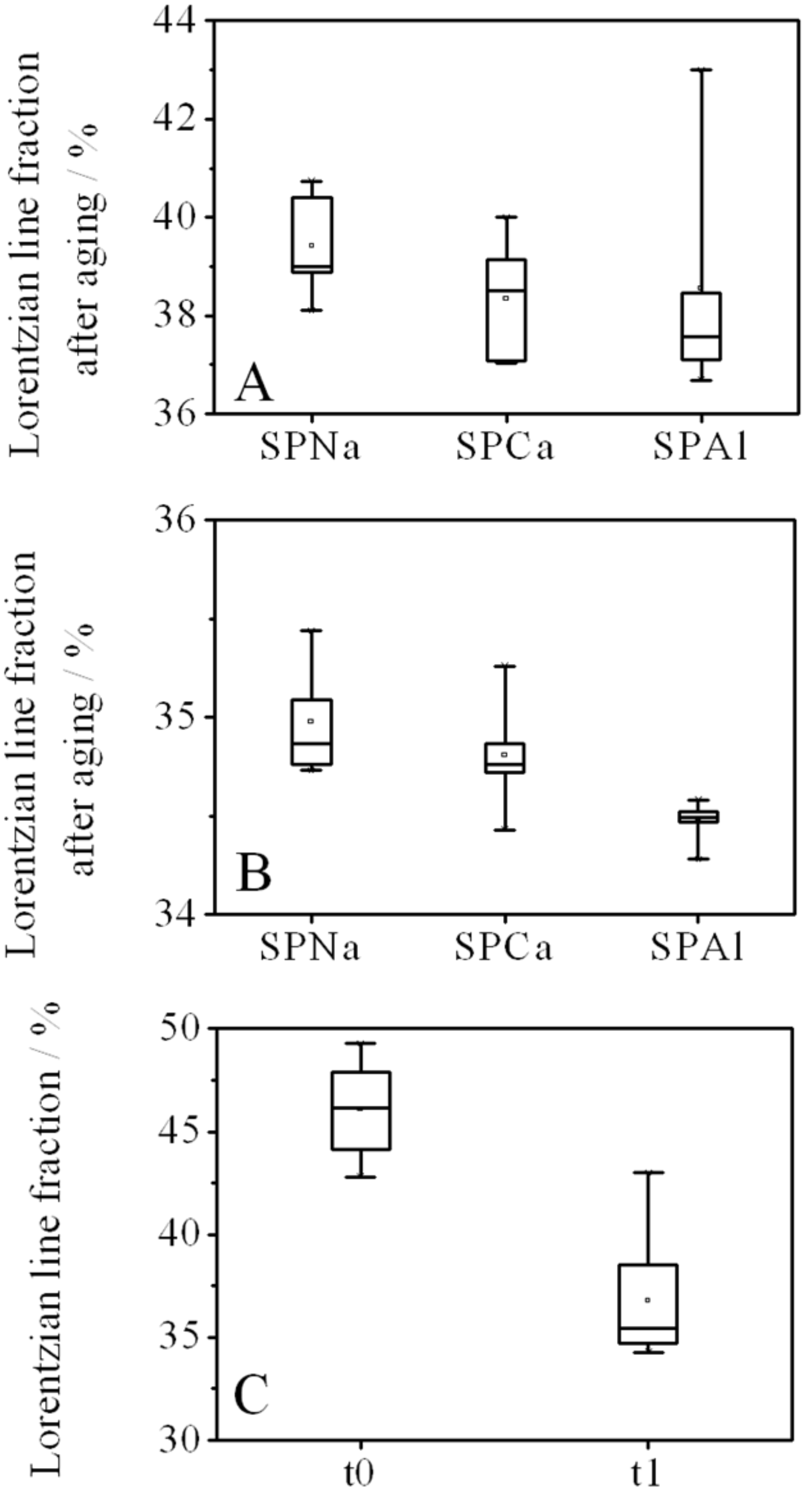

Figure S5. Lorentzian line fraction of ^{1}H wideline (amount of mobile water) after aging, shown with respect to cation types for SP-M@1.9 (A) and for SP-M@4.1(B). Lorentzian line fraction for all the treated samples before (t0) and after (t1) aging are shown in (C).
