# Supplementary material for: Restructuring of a Peat in Interaction with Multivalent Cations: Effect of Cation Type and Aging Time
Source: PLoS One. 2013 Jun 4;8(6):e65359. doi: 10.1371/journal.pone.0065359 (PMC3672098; doi:10.1371/journal.pone.0065359)
Supplement: Figure S6 — Change in amount of mobilisable water after aging for cation addition at pH 1.9 (A) and at 4.1 ((B), with respect to the loaded cation type. Dashed line along zero distinguishes the increase and decrease in amount of mobile water after aging. (PDF) [file pone.0065359.s006.pdf]

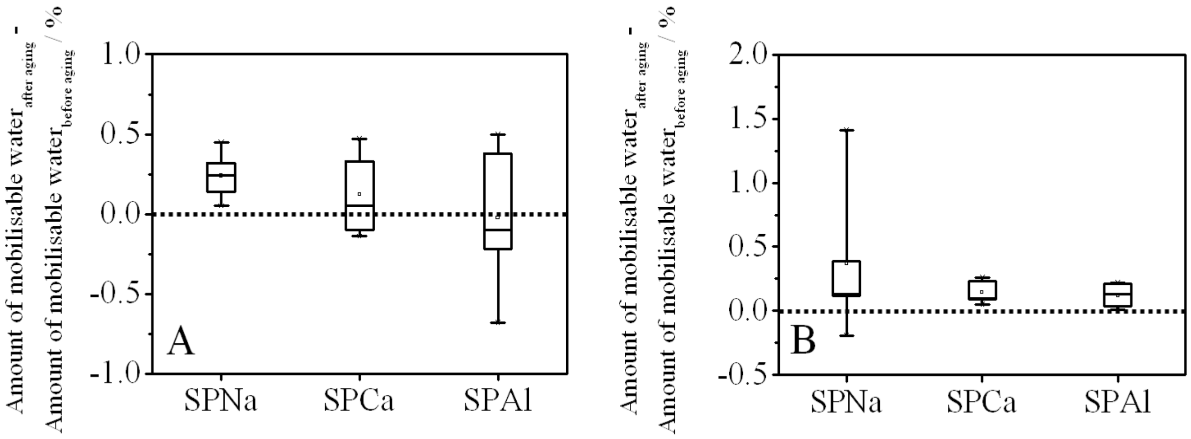

Figure S6. Change in amount of mobilisable water after aging for cation addition at pH 1.9 (A) and at 4.1 ((B), with respect to the loaded cation type. Dashed line along zero distinguishes the increase and decrease in amount of mobile water after aging.
