# Supplementary material for: Restructuring of a Peat in Interaction with Multivalent Cations: Effect of Cation Type and Aging Time
Source: PLoS One. 2013 Jun 4;8(6):e65359. doi: 10.1371/journal.pone.0065359 (PMC3672098; doi:10.1371/journal.pone.0065359)
Supplement: Table S1 — All the investigated parameters for untreated (SP), resin-treated (SP-H) and cation treated samples with different cations and cation concentration where cation treatment was carried out at pH 1.9. (PDF) [file pone.0065359.s007.pdf]

Sample	Amount of sorbed cation, M / $mmol_c kg^{-1}$	CEC_{eff} / $mmol_c kg^{-1}$	*DOC* / $mgkg^{-1}$	Contact angle /°	Step transition temperature, T* / °C	^{1}H NMR relaxation characteristics		^{1}H NMR wideline characteristics	
						$T_{2,fast}$ / ms	Contribution of fast-relaxing water to the total T_2 / %	Intensity of Lorentzian line, related to the total signal / %	Change in intensity of Lorentzian line after heating / %
SP		123 ± 12	2.3 ± 0.1	109 ± 3	56.0 ± 0.3	319 ± 16	12 ± 6	48.0 ± 0.2	2.1 ± 0.2
SP-H		256 ± 3	1.58 ± 0	127 ± 1	57.5 ± 0.5	150 ± 17	26 ± 3	44.5 ± 0.2	2.4 ± 0.2
SP-Al@1.9	2.5 ± 3.7	197.2 ± 3.4	1.1 ± 0	128 ± 5	55.3 ± 0.2	141 ± 17	24 ± 1	48.2 ± 0.2	2.0 ± 0.2
	2.8 ± 1.7	164.4 ± 2.1	1.6 ± 0	131 ± 2	55.9 ± 0.2	155 ± 8	26 ± 1	48.3 ± 0.2	1.7 ± 0.2
	2.8 ± 2.6	181.6 ± 2.2	1.1 ± 0	130 ± 5	55.6 ± 0.2	144 ± 1	26 ± 1	46.2 ± 0.2	2.4 ± 0.2
	3.3 ± 0.5	209.3 ± 1.7	1.1 ± 0.1	124 ± 2	55.2 ± 0.1	157 ± 2	23 ± 0	48.0 ± 0.2	2.6 ± 0.2
	5.4 ± 0.6	250.2 ± 3.1	1.34 ± 0	136 ± 1	57.1 ± 0.3	148 ± 1	23 ± 0	45.7 ± 0.2	3.2 ± 0.2
SP-Ca@1.9	5.9 ± 0.2	194.4 ± 1.0	1.5 ± 0	126 ± 1	55.2 ± 0.1	146 ± 7	25 ± 0	47.2 ± 0.2	2.1 ± 0.2
	6.6 ± 0.1	184.4 ± 3.1	2.1 ± 0.1	125 ± 2	55.4 ± 0.1	153 ± 0	23 ± 0	48.1 ± 0.2	2.3 ± 0.2
	6.6 ± 0.1	190.7 ± 8.9	1.5 ± 0	132 ± 2	55.9 ± 0.4	151 ± 2	24 ± 0	47.9 ± 0.2	2.2 ± 0.2
	6.2 ± 0.1	203.9 ± 1.6	1.6 ± 0.1	127 ± 2	55.6 ± 0.2	150 ± 2	22 ± 0	48.1 ± 0.2	2.2 ± 0.2
	5.7 ± 0.4	221.9 ± 2.6	1.6 ± 0	125 ± 3	56.6 ± 0.3	155 ± 1	21 ± 1	45.8 ± 0.2	2.1 ± 0.2
SP-Na@1.9	20.7 ± 3.4	167.6 ± 6.4	1.4 ± 0.1	121 ± 1	55.6 ± 0.2	158 ± 1	23 ± 0	49.3 ± 0.2	1.9 ± 0.2
	16.8 ± 2.4	139.8 ± 12	1.0 ± 0	120 ± 2	55.4 ± 0.2	165 ± 1	23 ± 1	46.7 ± 0.2	2.1 ± 0.2
	18.0 ± 0.9	163.0 ± 3.9	1.6 ± 0	131 ± 1	56.2 ± 0.1	156 ± 2	22 ± 1	48.4 ± 0.2	1.9 ± 0.2
	16.5 ± 1.3	196.6 ± 4.7	1.6 ± 0	129 ± 1	56.6 ± 0.5	169 ± 2	22 ± 0	47.4 ± 0.2	1.6 ± 0.2
	19.2 ± 0.8	216.9 ± 9.5	1.5 ± 0.1	128 ± 0	56.4 ± 0.2	150 ± 1	22 ± 1	46.1 ± 0.2	2.0 ± 0.2

Table S1. All the investigated parameters for untreated (SP), resin-treated (SP-H) and cation treated samples with different cations and cation concentration where cation treatment was carried out at pH 1.9.
