# Supplementary material for: Restructuring of a Peat in Interaction with Multivalent Cations: Effect of Cation Type and Aging Time
Source: PLoS One. 2013 Jun 4;8(6):e65359. doi: 10.1371/journal.pone.0065359 (PMC3672098; doi:10.1371/journal.pone.0065359)
Supplement: Table S2 — All the investigated parameters for cation treated samples with different cations and cation concentration where cation treatment was carried out at pH 4.1. (PDF) [file pone.0065359.s008.pdf]

Sample	Amount of sorbed cation, M / $mmol_c kg^{-1}$	CEC_{eff} / $mmol_c kg^{-1}$	*DOC* / $mgkg^{-1}$	Contact angle /°	Step transition temperature, T* / °C	^{1}H NMR relaxation characteristics		^{1}H NMR wideline characteristics	
						$T_{2,fast}$ / ms	Contribution of fast-relaxing water to the total T_2 / %	Intensity of Lorentzian line, related to the total signal / %	Change in intensity of Lorentzian line after heating / %
SP-Al@4.1	4.4 ± 0.4	214.4 ± 7.6	1.6 ± 0	139 ± 1	57.1 ± 0.1	73 ± 1	36 ± 0	46.8 ± 0.2	2.2 ± 0.2
	7.0 ± 0.2	57.4 ± 7.9	1.7 ± 0	130 ± 1	55.8 ± 0.1	72 ± 1	31 ± 2	43.7 ± 0.2	2.3 ± 0.2
	11.8 ± 0.3	113.9 ± 5.3	1.4 ± 0	134 ± 1	55.8 ± 0.2	75 ± 2	40 ± 2	43.7 ± 0.2	2.3 ± 0.2
	14.5 ± 0.1	62.9 ± 3.4	1.4 ± 0.1	135 ± 1	55.8 ± 0.1	79 ± 0	42 ± 0	45.4 ± 0.2	2.2 ± 0.2
	13.8 ± 1	216.8 ± 1.7	1.5 ± 0	127 ± 1	56.4 ± 0.3	82 ± 3	31 ± 4	45.6 ± 0.2	2.3 ± 0.2
SP-Ca@4.1	9.4 ± 4.7	243.5 ± 8.8	1.5 ± 0	133 ± 2	56.7 ± 0.1	61 ± 1	32 ± 6	43.3 ± 0.2	2.1 ± 0.2
	13.1 ± 1.3	181.0 ± 1.3	1.5 ± 0	132 ± 1	56.2 ± 0.1	58 ± 1	35 ± 2	43.8 ± 0.2	2.1 ± 0.2
	21.2 ± 2.6	198.3 ± 8.8	1.6 ± 0	122 ± 1	55.4 ± 0.1	62 ± 1	29 ± 1	47.6 ± 0.2	2.1 ± 0.2
	27.2 ± 2.5	184.8 ± 8.2	1.6 ± 0	125 ± 2	56.6 ± 0.2	71 ± 2	31 ± 3	43.6 ± 0.2	2.2 ± 0.2
	27.6 ± 8.2	201.8 ± 5.7	1.7 ± 0	128 ± 1	55.9 ± 0.1	67 ± 2	28 ± 3	42.9 ± 0.2	2. ± 0.2
SP-Na@4.1	25.7 ± 5.1	196.1 ± 1.2	1.7 ± 0	120 ± 2	55.5 ± 0.1	73 ± 1	32 ± 2	45.8 ± 0.2	2.4 ± 0.2
	30.5 ± 1.3	189.9 ± 2.8	1.7 ± 0	127 ± 1	54.3 ± 0.2	60 ± 1	37 ± 6	47.5 ± 0.2	1.0 ± 0.2
	37.7 ± 0.9	196.8 ± 2.7	1.6 ± 0	135 ± 1	56.5 ± 0.2	70 ± 1	29 ± 2	44.6 ± 0.2	2.2 ± 0.2
	37.7 ± 1.0	201.3 ± 3.8	1.7 ± 0	123 ± 1	56.8 ± 0.1	71 ± 4	34 ± 4	42.8 ± 0.2	1.9 ± 0.2
	38.5 ± 0.8	191.1 ± 4.3	1.7 ± 0	134 ± 1	55.8 ± 0.2	60 ± 1	30 ± 7	44.2 ± 0.2	2.1 ± 0.2

Table S2. All the investigated parameters for cation treated samples with different cations and cation concentration where cation treatment was carried out at pH 4.1.
